# Treatment of trigeminal and glossopharyngeal neuralgia in an adolescent: a case report

**DOI:** 10.1186/s40981-021-00465-5

**Published:** 2021-08-08

**Authors:** Aiko Maeda, Kenzo Araki, Chiaki Yamada, Shoko Nakayama, Kazuhiro Shirozu, Ken Yamaura

**Affiliations:** 1 Department of Anesthesiology and Critical Care Medicine, Kyushu University Hospital, 3-1-1 Maedashi Higashi-ku, Fukuoka City, Fukuoka, 812-8582 Japan; 2 Operating Rooms, Kyushu University Hospital, Fukuoka, Japan; 3 Department of Anesthesiology and Critical Care Medicine, Kyushu University Graduate School of Medicine, Fukuoka, Japan

**Keywords:** Adolescent, Combined hyperactive dysfunction syndrome, Trigeminal neuralgia, Glossopharyngeal neuralgia

## Abstract

**Background:**

Hyperactive dysfunction syndrome (HDS) refers to a constellation of symptoms developing from cranial nerve overactivity caused by neurovascular compression at the root entry or exit zone near the brainstem. Although the combined features of HDS are seen in the elderly, there are no reports of such cases in adolescents, to date.

**Case presentation:**

A 17-year-old male was diagnosed with right glossopharyngeal neuralgia and treated with microvascular decompression. He experienced new-onset right facial pain later and was diagnosed with right trigeminal neuralgia, which required prompt radiofrequency thermocoagulation of the right mandibular nerve. Follow-up in the third post-treatment year revealed the absence of symptom recurrence.

**Discussion:**

We report the treatment of a rare case of adolescent-onset combined HDS presenting as trigeminal and glossopharyngeal neuralgia. This report highlights the possibility of combined hyperactive dysfunction syndrome in younger age groups. It is crucial to establish a diagnosis early on for prompt management.

## Background

Hyperactive dysfunction syndrome (HDS) refers to a constellation of symptoms such as trigeminal neuralgia (TN), hemifacial spasm, and glossopharyngeal neuralgia (GPN) that results from cranial nerve overactivity [1–3]. Overall, the pain evoked by HDS is remarkably severe such that it impairs the quality of life. TN is characterized by recurrent unilateral brief electric shock-like pain triggered by innocuous sensory stimuli [4, 5], while GPN is characterized by pain that radiates to the posterior tongue, pharynx and/or unilateral ear precipitated by deglutition, coughing, talking, or yawning [6, 7]. This pathology is caused by cranial nerve compression by a vessel at the root entry or exit zone (REZ) near the brainstem, which excludes other possible etiologies, such as multiple sclerosis and brain tumors.

In extremely rare cases, patients may present with combined HDS (i.e., HDS with two or more features) [1–3]. Because single and combined HDS usually affects adults, it is not routinely considered a differential diagnosis when assessing children or adolescents with paroxysmal facial pain. Here, we describe a rare case of combined HDS presenting as TN and GPN in an adolescent. This report highlights the possibility of combined hyperactive dysfunction syndrome in younger age groups and the importance of early diagnosis and prompt management.

## Case presentation

A 17-year-old male with no medical history presented with recurrent pain in the right pharynx. Since there were no oral and dental abnormalities seen, he was referred to our pain management clinic for pain evaluation and treatment.

The patient had a well-defined unilateral area of severe pain from the root of the tongue on the right to the pharynx. Intense paroxysmal electrical pain lasting a few seconds to minutes occurred approximately 10 times a day following tongue movements and deglutition. There were no bradycardia or syncopal episodes suggestive of vasovagal attacks. The patient rated the pain as 8/10 on the numerical rating scale (NRS). Magnetic resonance imaging (MRI) with a sequence of constructive interference in steady state (CISS) showed the compression of the glossopharyngeal nerve by the posterior inferior cerebellar artery (PICA). Further, no abnormal findings were noted in the otorhinolaryngologic and other cranial nerve examinations. Moreover, there were no signs of Eagle’s syndrome. These findings were consistent with the diagnosis of GPN, which required carbamazepine administration. Although high-dose carbamazepine (gradually increased to 800 mg daily) was effective initially, it had only a transient beneficial efficacy. Moreover, daily administration of 5 mg baclofen was found to be ineffective. Given the lack of response to conservative treatment and the evidence of nerve compression on MRI, he was referred for microvascular decompression (MVD) 2 years later. Although the post-operative course showed cerebral venous sinus thrombosis, pain symptoms improved. He was discharged 1 month later without sequelae.

**Fig. 1:**
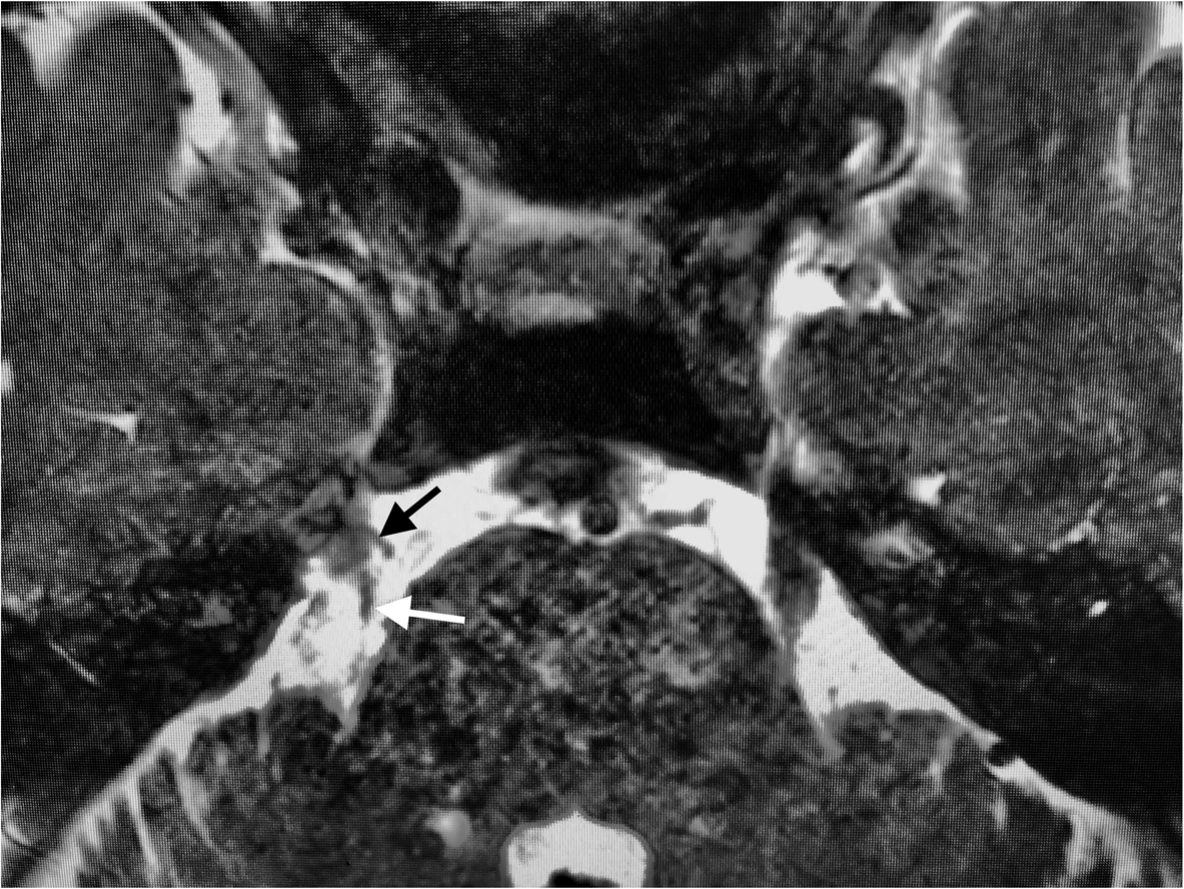
Axial magnetic resonance imaging with a sequence of constructive interference in steady state showing that the right trigeminal nerve was in contact with multiple vessels (white arrow: right superior cerebellar artery, black arrow; right petrosal vein)

**Fig. 2:**
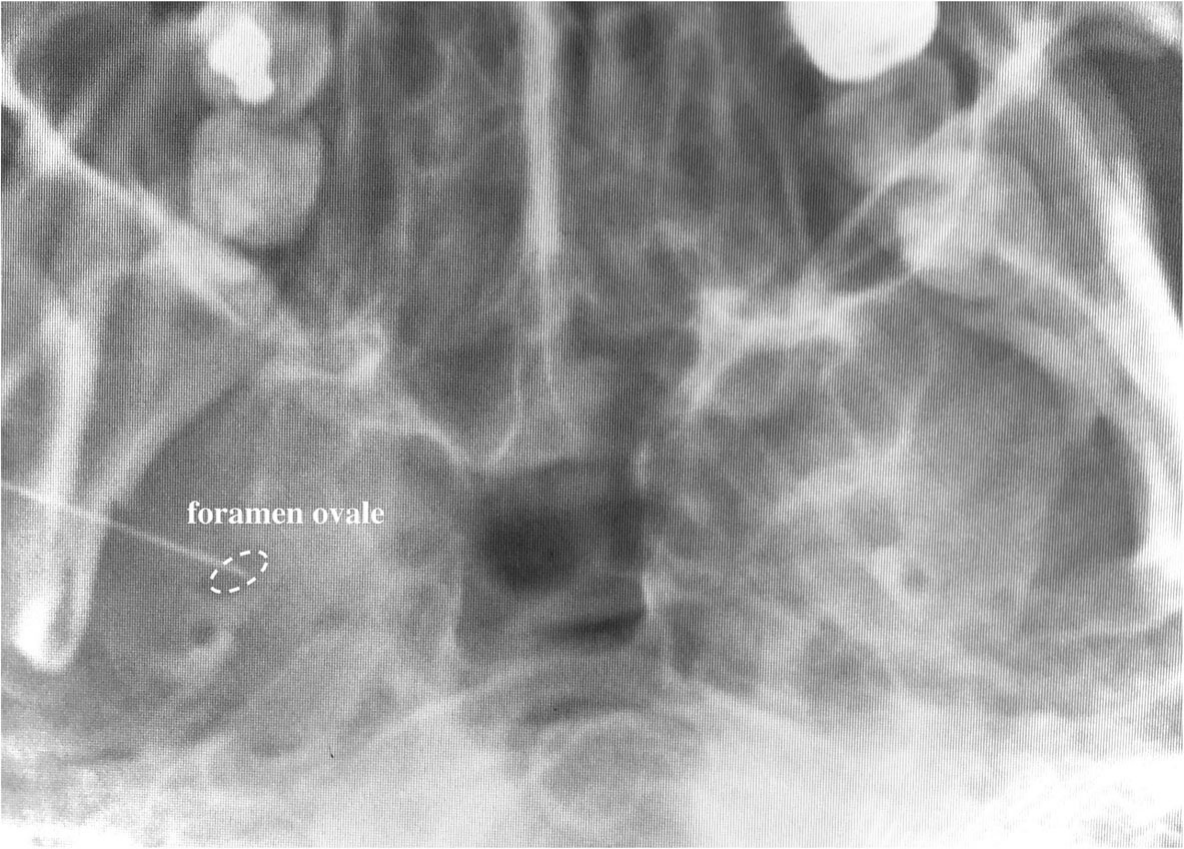
Fluoroscopic image of the right mandibular nerve during radiofrequency thermocoagulation. The needle tip is overlying the foramen ovale

One year later, he presented with a new-onset right facial pain. The pain was described as severe lancinating pain in the right preauricular region radiating to the mandible. The pain was worsened when lying or while chewing, and the maximum NRS was 8–10/10. These findings were consistent with the diagnosis of TN rather than the recurrence of GPN. Although carbamazepine (increased to 800 mg daily) improved his symptoms initially, his symptoms gradually deteriorated during the next few months. This significantly impaired his quality of life, such that he could not clean his teeth, chew and eat solid food, and sleep properly. The doses of pregabalin, duloxetine, and tramadol were gradually increased to 450 mg, 60 mg, and 300 mg daily, respectively, which yielded no response. CISS MRI revealed that the right trigeminal nerve was in contact with multiple right vessels, including the superior cerebellar artery (SCA) and petrosal vein (PV) (Fig. 1). Although the patient was deemed a good candidate for MVD, he chose to undergo a nerve block procedure instead. We decided to perform a minimally invasive treatment with radiofrequency thermocoagulation (RFT) of the right mandibular nerve. The patient was placed in the supine position with the head strongly bent backward using the shoulder pillow to visualize the foramen ovale on X-ray fluoroscopy. The puncture point was the midpoint of the zygomatic arch at the inferior border, approximately 3 cm ventral to the tragus. A 22-gauge 54-mm radiofrequency needle (TL-S; Top Corporation, Tokyo, Japan) was used to reach the foramen ovale, and 0.3 V sensory stimulation at 50 Hz was used to produce concordant pain. (Fig. 2). Within minutes of injecting 0.5 mL of 2% mepivacaine, sensory loss of the right mandible was obtained. Sequentially, RFT was performed for 90 s with 80 °C of heat (TOP Lesion Generator TLG-10; Top Corporation). Post-interventional course was uneventful without complications. Intractable pain in the right mandible ameliorated immediately after RFT, and his quality of daily life improved significantly. Although rare mild pain attacks had occurred within the next 3 years, these were well controlled by occasional oral administration of low-dose carbamazepine (200–400 mg daily).

This article adheres to the applicable Enhancing the Quality and Transparency of Health Research guideline. Written informed consent was obtained from the patient for publication of the case report.

## Discussion

Combined HDS is a rare condition that can possibly affect one or both sides either synchronously or metachronously. In 1998, Kobata et al. first described that combined HDS accounted for 2.8% of all patients with HDS and is accelerated by aging and hypertension [1]. Further, some recent studies have similarly revealed that combined HDS is present in approximately 3% of patients with HDS, and the mean age of patients with combined HDS was significantly higher than that of those with single HDS [2, 3]. To date, no study has reported on its development before 20 years of age. To the best of our knowledge, only case series and case reports of single HDS have been published in pediatric and adolescent populations [8–16]. In the present report, we present an extremely rare case of combined HDS in an adolescent patient.

HDS has been rarely mentioned in pediatric textbooks. In addition, the data from studies among the pediatric population reporting on TN and GPN suggested a high proportion of venous compression compared with adults, which might cause an atypical clinical presentation (e.g., pain that occurs while lying down at night) [10, 12]. Therefore, HDS in the young population is likely to cause a missed diagnosis or delayed appropriate treatment.

CISS MRI provides remarkable contrast between the cranial nerves, small vessels, and cerebrospinal fluid in the REZ; therefore, it is definitely useful in diagnosing HDS. Most cases of TN or GPN alone are usually caused by neurovascular compression at the REZ by meandering vessels. The most common offending vessels are the SCA, involving about 50% of TN cases, and the PICA in GPN, which are also responsible for cases of combined HDS [4–7]. However, not all cases of neurovascular compression are clinically symptomatic. Anatomical findings related to the transition zone between the central and peripheral myelin have suggested that the “transition zone” of the cranial nerves is susceptible to pressure from blood vessels [17, 18]. Therefore, the individual differences in the ‘transition zone’ of the cranial nerves may also be relevant to the HDS.

In the published literature, treatment of HDS in the pediatric and adolescent populations has been performed in the same manner as in elderly patients. Although medications are an effective first-line treatment option, when symptoms are refractory to them, surgical intervention is indicated. Many studies in the adult population reported the long-term efficacy of MVD. Pain relief was reported to be achieved in 73% of TN patients after 5 years, and in 76% of GPN patients after 2 years [4, 6]. Nevertheless, Bahgat et al. reported the characteristics, operative findings, and outcomes of TN patients aged ≤ 25 years. He identified that outcomes were not as good as those in the older population [19]. Several previous studies have reported that TN due to venous compression was recognized as a negative prognosis, which may be associated with MVD outcomes in such young patients [19, 20]. Also, fatal complications such as cranial nerve dysfunction (2%), stroke (0.3%), and death (0.2%) may occur. The second-choice neurosurgical treatment is gamma knife radiosurgery, which uses a focused radiation beam to sever the nerve root. However, recurrence rates were higher than in MVD [4–6]. Other surgical treatments include destruction in the ganglion or peripheral nerves, which can be performed chemically by glycerol blockade, mechanically by balloon compression, or thermically by RFT, with almost no major complications [4, 5]. RFT of the right mandibular nerve was performed for TN in our patient. This procedure, such as the approach to peripheral nerves or ganglion, can be used to achieve proper diagnosing of HDS. It is also an alternative therapy for MVD and easily performed at a low cost and can be repeated as necessary. However, the limitation is that it might be difficult for patients in younger age groups such as toddlers to cooperate during this procedure. Therefore, the utility and indications of these treatments for young patients require further investigations.

In conclusion, we report a very rare adolescent-onset combined HDS. GPN and TN were treated with MVD and RFT of the mandibular nerve, respectively, with no symptom recurrence for > 3 years to date. It is necessary to recognize that single and combined HDS may occur even in the pediatric or adolescent population. Symptom complaints might be atypical in pediatric–adolescent patients with HDS. Nevertheless, prompt diagnosis and management are crucial to improving patient outcomes.

## Data Availability

Not applicable

## Abbreviations

AICA: Anterior inferior cerebellar artery
CISS: Constructive interference in steady state
GPN: Glossopharyngeal neuralgia
HDS: Hyperactive dysfunction syndrome
MRI: Magnetic resonance imaging
MVD: Microvascular decompression
NRS: Numerical rating scale
PICA: Posterior inferior cerebellar artery
PV: Petrosal vein
REZ: Root entry or exit zone
RFT: Radiofrequency thermocoagulation
SCA: Superior cerebellar artery
TN: Trigeminal neuralgia
